# Exosomal miRNAs as Novel Pharmacodynamic Biomarkers for Cancer Chemopreventive Agent Early Stage Treatments in Chemically Induced Mouse Model of Lung Squamous Cell Carcinoma

**DOI:** 10.3390/cancers11040477

**Published:** 2019-04-04

**Authors:** Yu Zhou, Qi Zhang, Meijun Du, Donghai Xiong, Yian Wang, Altaf Mohammed, Ronald A. Lubet, Liang Wang, Ming You

**Affiliations:** 1 Center for Disease Prevention and Department of Pharmacology and Toxicology, Medical College of Wisconsin, Milwaukee, WI 53226, USA; peter.zhouyu@gmail.com (Y.Z.); qzhang@mcw.edu (Q.Z.); dxiong@mcw.edu (D.X.); yiwang@mcw.edu (Y.W.); 2 Department of Pathology, Medical College of Wisconsin, Milwaukee, WI 53226, USA; mdu@mcw.edu (M.D.); liwang@mcw.edu (L.W.); 3 Chemopreventive Agent Development Research Group, Division of Cancer Prevention, National Cancer Institute, Bethesda, MD 20892, USA; mohammeda2@mail.nih.gov (A.M.); lubetr@mail.nih.gov (R.A.L.)

**Keywords:** exosomal miRNAs, pharmacodynamic biomarkers, chemopreventive agent, lung squamous cell carcinoma

## Abstract

Background: Chemopreventive agent (CPA) treatment is one of the main preventive options for lung cancer. However, few studies have been done on pharmacodynamic biomarkers of known CPAs for lung cancer. Materials and methods: In this study, we treated mouse models of lung squamous cell carcinoma with three different CPAs (MEK inhibitor: AZD6244, PI-3K inhibitor: XL-147 and glucocorticoid: Budesonide) and examined circulating exosomal miRNAs in the plasma of each mouse before and after treatment. Results: Compared to baselines, we found differentially expressed exosomal miRNAs after AZD6244 treatment (*n* = 8, FDR < 0.05; *n* = 55, raw *p*-values < 0.05), after XL-147 treatment (*n* = 4, FDR < 0.05; *n* = 26, raw *p*-values < 0.05) and after Budesonide treatment (*n* = 1, FDR < 0.05; *n* = 36, raw *p*-values < 0.05). In co-expression analysis, we found that modules of exosomal miRNAs reacted to CPA treatments differently. By variable selection, we identified 11, 9 and nine exosomal miRNAs as predictors for AZD6244, XL-147 and Budesonide treatment, respectively. Integrating all the results, we highlighted 4 miRNAs (mmu-miR-215-5p, mmu-miR-204-5p, mmu-miR-708-3p and mmu-miR-1298-5p) as the key for AZD6244 treatment, mmu-miR-23a-3p as key for XL-147 treatment, and mmu-miR-125a-5p and mmu-miR-16-5p as key for Budesonide treatment. Conclusions: This is the first study to use circulating exosomal miRNAs as pharmacodynamic biomarkers for CPA treatment in lung cancer.

## 1. Introduction

Lung cancer continues to be the primary cause of cancer death in the U.S. and worldwide [1]. Chemopreventive agent (CPA) treatment is one of the main options for lung cancer prevention. The pharmacodynamic biomarkers of CPA treatments for lung cancers have been attracting the attention of scientists. Although some progress has been made, there is an urgent need for effective chemopreventive interventions and noninvasive methods of measuring chemopreventive efficacy (biomarkers) to eventually reduce lung cancer mortality. In this project, we aimed to examine the use of circulating exosomal microRNAs (miRNAs) as biomarkers to reflect the effect of CPAs in animal models of squamous cell carcinoma (SCC) of the lung.

miRNAs are small non-coding RNA molecules of 19–30 nucleotides in length that target protein-coding mRNA genes; miRNAs can inhibit gene expression by binding to complementary regions of mRNA and either block translation or degrade mRNA through the Argonaut complex [2,3]. Due to their influence on multiple genes involved in processes such as cell differentiation, development, cell death and homeostasis and fine-tuning the regulation of these pathways, cellular miRNA regulation has been implicated in malignant transformation. Subsequently, a set of miRNAs have been identified which may have a tumor suppressive role and could be useful in regressing tumors [4,5].

Extracellular vesicles such as exosomes and micro-vesicles are present in multiple biological fluids [6]. Exosomes are small (30–100 nm) membrane vesicles of endocytic origin that are released into the extracellular environment through fusion of multivesicular bodies with the plasma membrane [7]. Many cells, including tumor cells [8], have the capacity to release exosomes. On the one hand, these exosomes may enter the blood stream where their contents can be examined for diagnostic and prognostic applications. On the other hand, the transport of RNAs from tumor cells to neighboring cells and distant sites may have significant effects on tumorigenesis and metastasis. Tumor-derived exosomes play a role in transporting functional mRNA into recipient cells, leading to glioma cell proliferation, tumor growth and metastasis [9]. Release of let-7 miRNA via exosomes can deliver oncogenic signals and promote metastasis [10]. Collectively, these studies demonstrate critical role of exosomal miRNAs in cancer etiology and suggest exosomal miRNA as cancer diagnostic and prognostic markers.

Tumor-derived exosomes are detectable in the serum and plasma of patients with various tumor types [9,11,12,13]. For example, the circulating levels of tumor exosomes, exosomal small RNA and specific exosomal miRNAs in patients with and without lung adenocarcinoma were examined as biomarkers for diagnosis and prognosis in patients with adenocarcinoma of the lung [12]. Differences in exosomal miRNA levels were observed between lung cancer patients and controls and a significant similarity between the circulating exosomal miRNA and the tumor-derived miRNA patterns [12]. Thus, circulating exosomal miRNA may provide a powerful tool for noninvasive diagnosis of lung cancer and be particularly relevant to chemoprevention.

The aim of this study is to identify the circulating exosomal miRNAs as pharmacodynamic biomarkers of CPAs in mouse models of lung squamous cell carcinoma. To achieve the aim, we used a mouse lung SCC model by painting inbred strains of mice with N-nitroso-tris-chloroethylurea (NTCU) as previously described [14]. We treated the mice with three known CPAs (a MEK inhibitor AZD6244 [15], a PI3K inhibitor XL-147 [16] and a glucocorticoid Budesonide [17]). With the exosomal miRNA-seq data, we applied comprehensive methods to analyze the circulating exosomal miRNA change in each mouse before and after treatment. The results will facilitate understanding the complex chemopreventive mechanisms for lung cancer and applying specific exosomal miRNA signatures to select an appropriate chemopreventive regimen to prevent lung cancer and to regress the benign lesions.

## 2. Results

### 2.1. General Characteristics of Exosomal miRNA-seq Data

**Figure 1:**
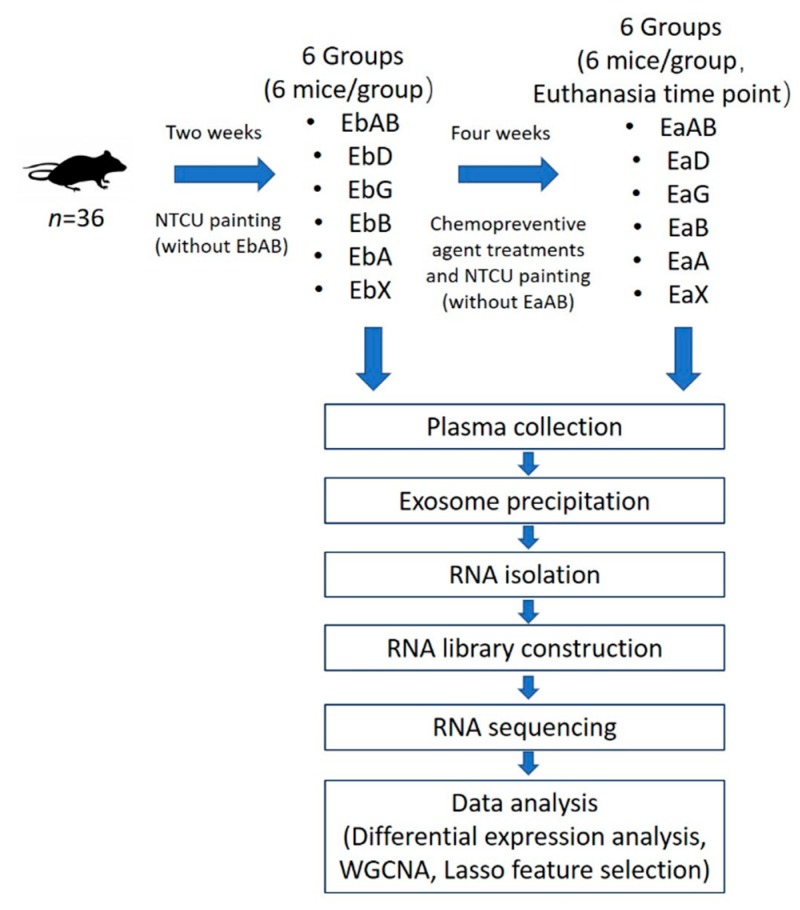
Flowchart of the experimental design. Among 36 mice tested in the study, except for 6 mice in absolute control group, the other 30 mice were painted with NTCU (N-nitroso-tris-chloroethylurea) for two weeks. The 30 mice with NTCU painting were randomly divided into five groups. Plasma from each mouse was collected before and 4 weeks after agent-specific treatment. Exosomal miRNAs from plasma were isolated and sequenced. Comprehensive analysis was performed to find the key exosomal miRNAs as novel pharmacodynamic biomarkers for cancer chemopreventive agent early stage treatments in chemically induced mouse model of lung squamous cell carcinoma. (EaA: early treatment after AZD6244; EaAB: early treatment after absolute control; EaB: early treatment after budesonide; EaD: early treatment after diet control; EaG: early treatment after early gavage control; EaX: early treatment after XL-147; EbA: early treatment before AZD6244; EbAB: early treatment before absolute control; EbB: early treatment before budesonide; EbD: early treatment before diet control; EbG: early treatment before early gavage control; EbX: early treatment before XL-147).

Figure 1 and Table 1 showed the pipeline and details of this study design. For each exosomal miRNA library, we obtained an average of 10,089,414 raw reads (10,089,414 ± 2,357,990). Approximately 50% raw reads (5 million reads) were mapped to known miRNA. We further grouped the exosomal miRNA sequence data into different groups and found no obvious sequencing read counts difference between control and treatment groups. For further analysis, we excluded those miRNAs with log_2_ transformed read counts <3. From the 72 exosomal miRNA sequencing libraries, we detected 207 miRNAs with log2 transformed read counts >3. The five most abundant miRNAs included mmu-miR-128-3p, mmu-miR-148a-3p, mmu-miR-99a-5p, mmu-let-7i-5p and mmu-let-7b-5p (log_2_ transformed read counts were 18.0, 17.2, 16.1, 15.3 and 15.1, respectively, and Table S1).

### 2.2. Differentially Expressed (DE) Exosomal miRNAs by CPA Treatments

**Figure 2:**
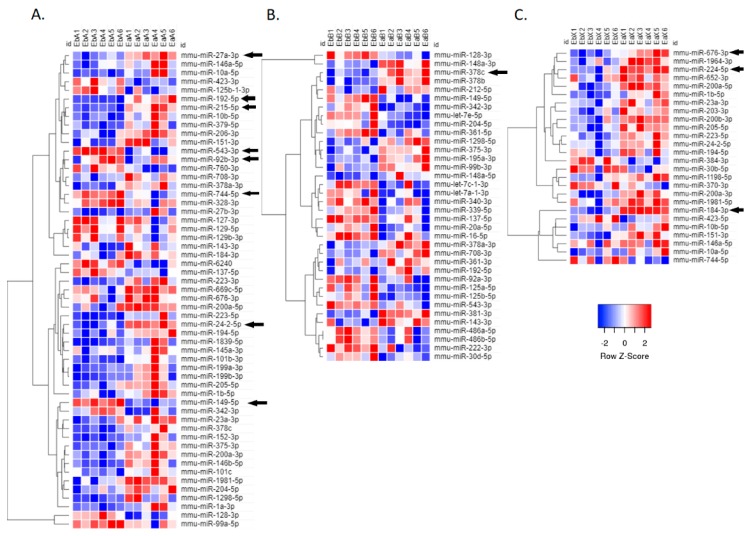
Exosomal miRNA expression affected by chemopreventive agent treatments. (**A**) Heat map showing differentially expressed exosomal miRNA affected by AZD6244 treatment. (**B**) Heat map showing differentially expressed exosomal miRNA affected by Budesonide treatment. (**C**) Heat map showing differentially expressed exosomal miRNA affected by XL-147 treatment. Expression values are shown as log2 transformed read counts and the criterion is raw *p*-value < 0.05. The significant differentially expressed miRNAs shown in Table 2 (FDR < 0.05) are labeled by arrows. Low expression is indicated by blue, and high expression is indicated by red.

Based on the high stringent *p*-values after adjustment by a false-discovery-rate (FDR) based multiple-test correction (FDR < 0.05), eight exosomal miRNAs were differentially expressed after AZD6244 treatment of which four exosomal miRNAs were down-regulated and four exosomal miRNAs were up-regulated (Table 2). At the same stringent differential expression criterion, only four exosomal miRNAs were differentially expressed after XL-147 treatment (all up-regulated; Table 2) and one exosomal miRNAs were differentially expressed after Budesonide treatment (up-regulated; Table 2). However, when the criterion was relaxed to raw *p*-value < 0.05, 55 (AZD6244 treatment), 26 (XL-147 treatment) and 36 (Budesonide treatment) exosomal miRNAs were differentially expressed (Tables S2–S4 and Figure 2). In the comparison among these nominal DE exosomal miRNAs, 16 miRNAs were overlapped between AZD6244 and XL-147 treatments and their fold change directions were the same. Between AZD6244 and Budesonide treatments, 13 miRNAs were overlapped, and the fold change directions were the same. However, the fold changes after Budesonide treatment were relatively small (absolute log_2_ fold change less than 1). There were no overlapped miRNAs between XL-147 and Budesonide treatments.

### 2.3. Exosomal miRNAs Co-expression Network Modules

**Figure 3:**
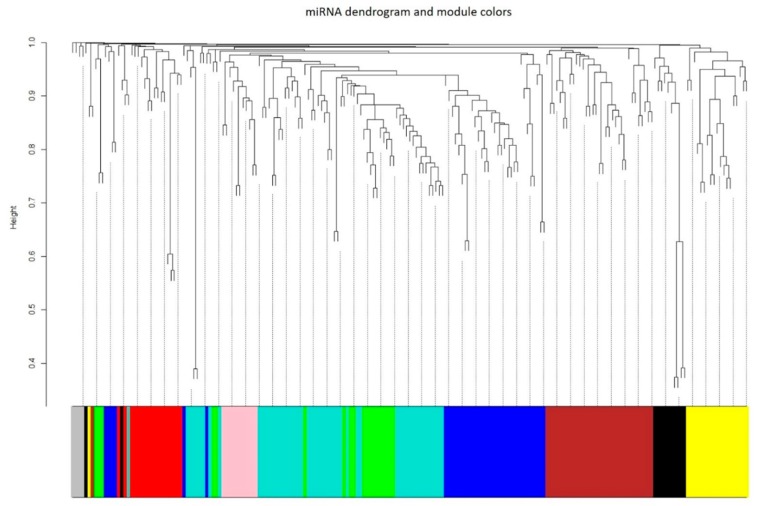
Exosomal miRNA co-expression network analysis by WGCNA. Hierarchical cluster dendrogram of the 207 identified exosomal miRNAs. Modules corresponding to branches are labeled with colors indicated by the color bands underneath the tree. Number in each cell shows the correlation of the corresponding module eigengene and spontaneous lung cancer susceptibility, with the *p*-value printed below the correlation. The cell color was coded by correlation according to the color legend.

**Figure 4:**
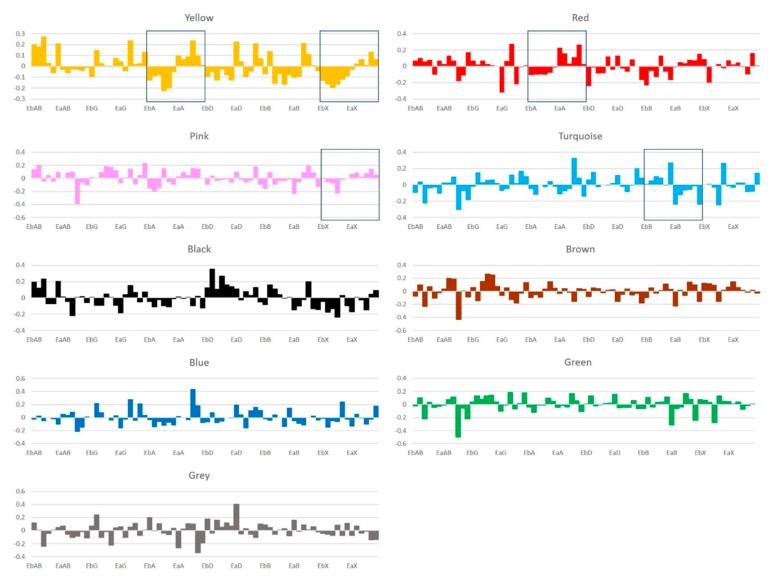
Module-sample relationships. Each bar corresponded to a module eigengene in a sample. Module eigengenes (MEs) were defined as the first principal component calculated using PCA, which can summarize modules’ behavior. Yellow module was up-regulated in all the mice in AZD6244 and XL-147 treatment groups. Red module was up-regulated in all the mice in AZD6244 treatment group. Pink module was up-regulated in all the mice in XL-147 treatment group. Turquoise module got down-regulated in all the mice in Budesonide treatment group.

From the heatmap of exosomal miRNA expression levels, we observed multiple patterns of co-expression across the samples. Therefore, we applied Weighted Gene Co-expression Network Analysis (WGCNA) to cluster the exosomal miRNAs into modules based on their paired correlation. Figure 3 showed a total of nine modules that were labeled by different colors (exosomal miRNAs in modules: black, 12; blue, 37; brown, 34; green, 19; grey, 4; pink, 11; red, 18; turquoise, 52; yellow, 20). To further analyze the effect of each CPA on different module, module eigengenes (MEs) for each sample were calculated. ME was the first principal component calculated via PCA to represent the all miRNAs in a module. Figure 4 showed the ME profiles of each sample. In Figure 4, yellow module exhibited common patterns of change in both AZD6244 treated and XL-147 treated mice. The results were consistent with the differential expression analysis. The most shared DE miRNAs (total *n* = 16) between AZD6244 and XL-147 treatments were assigned into yellow (*n* = 8) module. Meanwhile, MEs of red module were up-regulated in all AZD6244 treated mice, suggesting that miRNAs in the red module might be involved in the biological function that was specific to AZD6244 treatment. Similarly, the pink module was only up-regulated in the mice of XL-147 treatment. Responding to Budesonide treatment, turquoise module was unique and down-regulated in each treated mouse.

### 2.4. Signatures of Exosomal miRNA Expression Change after CPA Treatments

In co-expression network analysis, several modules showed specific response to different CPA treatments, like red module to AZD6244 treatment, pink module to XL-147 treatment and turquoise module to Budesonide treatment. Least absolute shrinkage and selection operator (Lasso) method was performed to determine whether the subset of exosomal miRNAs could be used to predict particular CPA treatment. Using the fold change of each exosomal miRNA between before- and after- treatment, we identified 11, 9 and 9 exosomal miRNAs as signatures for predicting AZD6244, XL-147 and Budesonide treatment, respectively (Table S5). Then we combined the results from differential expression analysis, co-expression analysis and signature selection analysis. We screened the exosomal miRNAs to find key ones with following criterions: (1) Differentially expressed after CPA treatment (raw *p*-value < 0.05); (2) Be included in the co-expression modules responding to specific CPA treatment (red module for AZD6244 treatment, pink module for XL-147 treatment and turquoise module for Budesonide treatment); (3) Be selected as predictors for specific CPA treatment by lasso method. As a result, we detected that 4 DE exosomal miRNAs (mmu-miR-215-5p, mmu-miR-204-5p, mmu-miR-708-3p and mmu-miR-1298-5p) distinguished the AZD6244 treatment group. For XL-147 treatment, we found one DE exosomal miRNA (mmu-miR-23a-3p) in pink module as a part of signature. Meanwhile, two DE exosomal miRNAs (mmu-miR-125a-5p and mmu-miR-16-5p) in turquoise module were considered as predictors for Budesonide treatment.

### 2.5. Pathway Enrichment Analysis

To further understand the function of exosomal miRNAs affected by CPA treatments, we used mirPath to decipher these microRNA functions. The predicted target genes of four key exosomal miRNAs for AZD6244 treatment were mostly enriched in Estrogen signaling pathway (mmu04915) (Table 3). In this pathway, several predicted target genes, like Shc1, Sos1 and Atf2, were also the essential genes of MAPK/ERK signaling pathway that was inhibited by AZD6244 treatment. For XL-147 treatment, since there was only one key miRNA, we only screened the potential target genes of mmu-miR-23a-3p. Pik3r3 was predicted to be the target of mmu-miR-23a-3p. Meanwhile, another important gene in Pi3k signaling pathway, Pik3c2a, was proved to be regulated by mmu-miR-23a-3p by immunoprecipitation experiment [18]. For Budesonide treatment, the most enriched pathway of predicted target genes was Galactose metabolism (mmu00052) that was closely related to glucocorticoid treatment (Table 4).

### 2.6. Conservation Score Analysis

We compared the key exosomal miRNAs found above with the candidate homologs in human beings. The sequences of five key mature miRNAs (mmu-miR-204-5p, mmu-miR-708-3p, mmu-miR-23a-3p, mmu-miR-125a-5p and mmu-miR-16-5p) were same between mouse and human beings. For mmu-miR-215-5p and mmu-miR-1298-5p, only one nucleic acid was mismatched between the homologs in mouse and human beings.

## 3. Discussion

To our best knowledge, this is the first study to determine the effects on exosomal miRNA expression of three known CPAs in mouse models of lung SCC and develop circulating exosomal microRNAs as biomarkers for specific CPA treatment. We applied a series of analytical approaches to select the most promising exosomal miRNAs which were detectable, representative and distinguished for each agent. At least, when the threshold was relaxed to raw *p-*value < 0.05, the differential expressions of key miRNAs were significant.

In differential expression analysis, 16 miRNAs (raw *p-*value < 0.05) were shared between DE miRNA lists of AZD6244 and XL-147 treatment and most of these miRNAs (15 of 16 miRNAs) were up-regulated after agents’ treatment. Interestingly, a study has shown that MAPK/ERK (inhibited by AZD6244) and PI3K (inhibited by XL-147) signaling pathways had cross-talk with each other [19]. Multiple downstream genes, like Bcl-2-associated death promoter (BAD) and glycogen synthase kinase 3 (GSK3) were regulated by both MAPK/ERK and PI3K signaling pathways [19]. Turke et al. also found that MEK inhibition led to PI3K/AKT activation via ERBB receptors [20]. Meanwhile, 13 miRNAs were found to be differentially expressed for both AZD6244 and Budesonide treatment and the regulation directions were same. Budesonide is a glucocorticoid used as an anti-inflammatory agent and one of mechanisms that glucocorticoid exert anti-inflammatory effects is to inhibit MAPK/ERK pathway [21,22]. However, although there were evidences showing that glucocorticoid was associated with PI3K pathway [23,24,25], we did not find overlapping DE miRNAs between XL-147 and Budesonide treatment. These results suggested unique co-expressed exosomal miRNA networks when mice were treated by different CPAs. It was supported by our WGCNA results. In cluster analysis, yellow module was up-regulated in each mouse after treated with AZD6244 or XL-147 and contained eight of 16 shared DE miRNAs between AZD6244 and XL-147 treatment. As expected, the target genes of these eight miRNAs were mostly enriched in Thyroid hormone signaling pathway (mmu04919) that included the major parts of MAPK/ERK and PI3K signaling pathways. 

However, when we tried to analyze the functions of these modules, we met a challenge that each module included tens of miRNAs and many miRNAs had hundreds of predict target genes. Either the pathway analysis or function analysis, like gene ontology terms, generated too many false positive results. To address this issue, we applied a feature selection method, Lasso, to further determine the exosomal miRNAs distinguishing different CPA treatments. Then, we compared the selected features with DE list and WGCNA results. We found that 4 DE exosomal miRNAs (mmu-miR-215-5p, mmu-miR-204-5p, mmu-miR-708-3p and mmu-miR-1298-5p) in red module were selected to be the predictors for AZD6244 treatment. miR-215 was reported to be a tumor suppressor in human non-small cell lung cancer by targeting ZEB2 [26] that could be regulated via MAPK/ERK pathway [27]. For miR-204-5p, Ye et al. found that in cancers of respiratory system miR-204-5p level was significantly decreased in lung cancer tissues compared with normal tissues (both *p* < 0.05) [28] via a comprehensive exploration based on RNA-seq high-throughput data and bioinformatics. miR-204 might also inhibit STAT3 and favor the MAPK signaling pathway in cutaneous squamous cell carcinoma progression [29]; miR-708-3p was found to inhibit breast cancer cell epithelial-to-mesenchymal transition (EMT) by directly binding to ZEB1 [30] which was also the downstream gene of MAPK/ERK pathway [31]; miR-1298 was also found to inhibit mutant KRAS-driven tumor growth [32]. MAPK signaling was downstream pathway of KRAS activation. These evidences supported our results for AZD6244 treatment. AZD6244 was a CPA and the four exosomal miRNAs which were tumor suppressors were up-regulated after treatment. The target genes of these four exosomal miRNAs were also associated with MAPK/ERK pathway.

For Budesonide treatment, mmu-miR-125a-5p and mmu-miR-16-5p were down-regulated. The previous studies showed that miR-125a-5p and miR-16-5p would induce apoptosis via activating p53 [33,34]. Glucocorticoid receptor activation might inhibit p53-induced apoptosis [35,36]. These results agreed with the negative association between mmu-miR-125a-5p/mmu-miR-16-5p and Budesonide (Glucocorticoid) treatment. However, the results were puzzled for XL-147. XL-147 was also a CPA for lung cancer and mmu-miR-23a-3p was overexpressed after XL-147 treatment. But miR-23a-3p was up-regulated in non–small cell lung cancer [37] and promote tumor growth and suppress apoptosis in gastric cancer [38]. A further investigation is required to clarify the relationship between XL-147 and mmu-miR-23a-3p.

In pathway analysis, the target genes of the key exosomal miRNAs were found to be enriched in several pathways. And the results were consistent with the findings of previous studies. For the top three enriched pathways of AZD6244 treatment, there was a strong cross-talk effect between MEK and estrogen receptor signaling pathway [39] and the AZD6244 treatment was verified to reverse antiestrogen resistance in ovarian cancer [40,41]. Although there was no study showing the AZD6244 treatment effect on adrenergic signaling in cardiomyocytes, another MEK inhibitor, TAK733, was just reported to attenuates adrenergic receptor-mediated cardiomyocyte hypertrophy via inhibiting Erk_Thr188_ phosphorylation [42]. The AMP-activated protein kinase (AMPK) could be activated by the combined usage of the MEK inhibitor selumetinib (AZD6244) and the AKT inhibitor MK2206 [43]. For the top three enriched pathways of Budesonide treatment, Budesonide treatment would induce upregulation of Mucin 1 and Mucin 4 [44] which carry up to five and six O-glycans, respectively [45]. Glucocorticoid treatment would also affect galactose metabolism [46] and enhance induced pluripotent stem cell reprogramming [47]. Nevertheless, we realized that the limited number of miRNAs in enrichment analysis may generate false positive interpretation. Future analysis is needed to validate the finding.

There were some limitations of our study. First, since our research was a pilot study using exosomal miRNAs as novel pharmacodynamic biomarkers for cancer chemopreventive agent, we could not find similar studies in human beings. Second, the function studies on these key exosomal miRNAs were mainly using human cells and xenograft mice model. To demonstrate the potential function of these key exosomal miRNAs, we screened these miRNAs for the conservation scores using miRviewer [48]. All the scores in mouse were over 0.8. It suggested that these exosomal miRNAs might have similar function in mouse and human. The chemopreventive agents used in this study were not specific for the mouse. The chemopreventive agents used in this study were not specific for the mouse. Edmund Poon et al. [49] showed that AZD6244 could inhibit the MEK pathway in CT26 mouse syngeneic model and Hung Huynh et al. [50] reported the same function of AZD6244 in human tumor cell lines and Xenograft model. Benedikt Bosbach et al. [51] treated tumor lysates from *Kit^V558Δ/+^* mice with XL147 for 4 h and found decreased PI3K signaling with reduced pAKT, pS6, and p4EBP1. Similar effects of XL147 were also identified in human tumor cell lines and Xenograft model [16]. For budesonide, it has been clinically used in the treatment of skin and respiratory disease [52,53] and S Edsbäcker et al. found that rates and routes of budesonide metabolism were most similar in mouse and human livers [54].

In summary, this is the first study to use exosomal miRNAs as pharmacodynamic biomarkers for three CPAs (AZD6244, XL-147 and Budesonide). In this study, we used lung SCC mice model and found that differentially expressed exosomal miRNAs after CPA treatments were partially co-expressed. We also found that several co-expressed modules were specifically responding to unique CPA treatment. By further feature selection, we highlighted mmu-miR-215-5p, mmu-miR-204-5p, mmu-miR-708-3p and mmu-miR-1298-5p as the key exosomal miRNAs for AZD6244 treatment, mmu-miR-23a-3p as key for XL-147 treatment and mmu-miR-125a-5p and mmu-miR-16-5p as key for Budesonide treatment. These results may facilitate understanding the complex chemopreventive mechanisms for lung cancer.

## 4. Materials and Methods

### 4.1. Reagents and Animals

Animal procedures were in accordance with the Medical College of Wisconsin Institutional Animal Care and Use Committee. In order to establish the lung SCC model, N-nitroso-tris-chloroethylurea (NTCU) was used. The NTCU was purchased from Toronto Research Chemicals, Inc. (Toronto, ON, Canada). Eight-week-old NIH Swiss mice received NTCU treatment through repeated skin painting as described previously in our publications [14,55,56,57,58]. Specifically, NTCU treatment was started when mice were ~8 weeks of age. The dorsal skin of each mouse was shaved prior to NTCU treatment. The NTCU was applied to the shaved dorsal skin of each mouse in 100-microliter (μL) drops of 0.04 M NTCU with a Gilson 200 μL micro-pipette. This process was repeated twice a week with a 3.5-day interval during the whole study (6 weeks). The body weight of each mouse was taken weekly for the duration of treatments.

One week after the first dose of NTCU treatment, mice were divided into the various groups listed in Table 1. The mice were treated with the indicated agent beginning two weeks after the first dose of NTCU and the treatments continued for four consecutive weeks. The treatment groups included budesonide, AZD6244, and XL-147. The dosage for budesonide was 1.5 mg/kg in diet. The dosage for AZD6244 was 40 mg/kg body weight. The dose for XL-147 was 100 mg/kg body weight. AZD6244 and XL-147 were freshly prepared in Mazola corn oil. Mice were treated once a day, 5 days a week via oral gavage with an 18-gauge gavage needle, 0.2 mL per mouse. Gavage Control animals were treated with 0.2 mL corn oil throughout the study. Diet control mice were feed with AIN-76A Purified Diet #100000 (Dyets Inc., Bethlechem, PA, USA) for the duration of the study, with or without oral gavage with vehicle. Mice were euthanized by CO_2_ asphyxiation at the indicated time points. Plasma was collected by retro-orbital bleeding before CPAs’ treatment as “pre-treated” or after CPAs’ treatment as “after-treated” plasma. Plasma was stored in a −80 °C freezer until use. The detailed time points are showed in Figure 1.

### 4.2. Exosome Precipitation and RNA Isolation

After collecting all the plasma samples, we thawed plasma and centrifuged at 3000× *g* for 15 min to remove remaining cells and cell debris. We then mixed 120 μL plasma with appropriate volume of ExoQuick reagents for overnight at 4 °C. The mixture was centrifuged at 1500× *g* for 30 min. The exosome pellet was dissolved in 25 μL 1× PBS (pipetting up and down multiple times, to make sure the pellet was suspended completely). The 25 μL of exosome suspension was mixed with 700 μL QIAzol lysis buffer, mixed well by pipetting and vertexing until there were no more white clumps. RNA was extracted according to the manufacturer’s standard protocol with DNase I treatment on column (miRNeasy Micro Kit, QIAGEN, Valencia, CA, USA). The extracted RNA was eluted with 15 μL of RNase-free water. The quantity and quality of the RNAs were checked by Agilent Bioanalyzer 2100 with a Small RNA Chip (Agilent Technologies, Santa Clara, CA, USA).

### 4.3. RNA Library Preparation and Sequencing

The RNA libraries were prepared following the instructions of the standard protocol [59] (NEBNext Multiplex Small RNA Library Prep Set, NEB, Ipswich, MA, USA). For each library, about 3–6 μL of miRNA was used as input RNA. Each library was prepared with a unique indexed primer (Index set1 and set2, NEB) and amplified for 15 cycles. The amplified libraries (DNA) were purified by using 1.8× AMPure beads, diluted into 27 μL ddH20, and qualified by Agilent High Sensitivity DNA Chips (Agilent Technologies, Santa Clara, CA, USA).

A total of 18–24 RNA libraries were pooled with equal molar concentration of the target band. Pooled libraries were resolved on 5% Mini-Protean Precast Gels (Bio-Rad, Hercules, CA, USA). DNA fragments from 146–164 bp (the length of miRNA inserts plus the 3′ and 5′ adaptors) were recovered in 12 μL of elution buffer (QIAGEN, Valencia, CA, USA). The indexed library pools were checked for quality and quantified by Agilent High Sensitivity DNA Chip; 10 μL of eight library pools (one flow cell) at a final concentration of 2 nM were sent to the Core Facility at Medical College of Wisconsin for 50 bp single read sequencing using Illumina HiSeq2000 DNA sequence analyzer (Illumina, Inc., San Diego, CA, USA).

### 4.4. Sequencing Data Analysis

#### 4.4.1. Differential Expression Analysis

The RNA-seq data (fastq files) were mapped by using DNASTAR SeqMan against the mouse miRNA sequences downloaded from miRBase 21 (http://www.mirbase.org/) [60,61,62,63,64]. The sequence counts that mapped to miRNAs were normalized as read count per million (read counts of an individual miRNA/sum of read counts of all mappable miRNAs × 10^6^). The miRNAs with log_2_ transformed read counts <3 were excluded. Since we collected the “pre-treated” and “after-treated” plasma from each mouse, paired *t*-test was used to detect if there were significantly differential expression levels of exosomal miRNAs after the CPA treatments. The Benjamini-Hochberg procedure was used to calculate the false discovery rate.

#### 4.4.2. Weighted Gene Co-expression Network Analysis

In differential expression analysis, we found that exosomal miRNAs exhibit multiple patterns of co-expression across the samples. We then used WGCNA R package to cluster exosomal miRNAs into modules with correlated patterns of variation [65]. First, we calculated Pearson correlation coefficients for all miRNA–miRNA comparisons across mouse plasma samples. Then, the matrix of correlations was converted to an adjacency matrix of connection strengths. Based on the adjacency matrix, modules were defined as sets of miRNAs with high topological overlap using the methods previously outlined by Zhang and Horvath [65,66]. Module eigengenes (MEs) were defined as the first principal component calculated using principal component analysis (PCA), which could summarize modules’ behavior.

#### 4.4.3. Least Absolute Shrinkage and Selection Operator

Glmnet R package [67] was used to identify the miRNA signature for CPA treatments. Glmnet was a package that fits a generalized linear model via penalized maximum likelihood [67]. The basic concept of generalized linear model was to assign a coefficient (β) to each independent variable (*x*) to predict the dependent variable (*y*). In our case, *x* was the fold change of each miRNA after CPA treatments and *y* was binary trait for each CPA treatment. For example, when we select signatures for AZD6244 treatment, the 6 AZD6244 treated mice were labelled as “*y* = 1” and the other 30 mice were labelled as “*y* = 0”. We used least absolute shrinkage and selection operator (Lasso) [68] regression implemented in Glmnet package to generate the prediction signature. We used the Glmnet package to randomly divide the sample dataset into 10 folds and performed cross-validation to generate the optimal λ for the prediction model which gave minimum mean cross-validated error.

#### 4.4.4. Molecular Pathway Analysis

For miRNA-based molecular pathway analysis of important chemoprevention associated miRNAs, we used DIANA-mirPath V.3 [69], a web-based application to perform an enrichment analysis of the predicted target mouse genes. The combinatorial effect of co-expressed miRNAs in the modulation of a given pathway were also addressed by DIANA-mirPath V.3 through the simultaneous analysis of multiple miRNAs. miRNA target genes implicated in a given pathway were graphically annotated on the pathway map providing a direct overview of the miRNA modulated parts, facilitating the interpretation and presentation of miRNA-dependent regulation of biological pathways. After supplying DIANA-mirPath V.3 with a list of miRNA names that we were interested in, it retrieved those miRNA target genes by using the miRNA target prediction programs: DIANA-microT V5.0 [70,71,72]. DIANA-mirPath V.3 performed an enrichment analysis of the input datasets by comparing each set of miRNA-target genes to all available biological pathways provided by the Kyoto Encyclopedia of Genes and Genomes (KEGG) [73]. All the default parameters were applied and adjusted *p-*value (FDR) less than 0.05 was used as threshold.

#### 4.4.5. Conservation Analysis

To compare the key exosomal miRNAs we found in mouse model with the homologous ones in human beings. We screened these miRNAs for the conservation scores using miRviewer [48].

## 5. Conclusions

In this study, we treated mouse models of lung squamous cell carcinoma with three different CPAs (MEK inhibitor: AZD6244, PI-3K inhibitor: XL-147 and glucocorticoid: Budesonide) and screened the expression level changes of exosomal miRNAs responding to CPAs treatment. Using comprehensive analysis methods, we highlighted four miRNAs (mmu-miR-215-5p, mmu-miR-204-5p, mmu-miR-708-3p and mmu-miR-1298-5p) as the key for AZD6244 treatment, mmu-miR-23a-3p as key for XL-147 treatment, and mmu-miR-125a-5p and mmu-miR-16-5p as key for Budesonide treatment. This is the first study to use circulating exosomal miRNAs as pharmacodynamic biomarkers for CPA treatment in lung cancer. Our results not only reveal key exosomal miRNAs as predictive biomarkers for CPAs but also facilitate understanding the complex chemopreventive mechanisms for lung cancer.

## Figures and Tables

**Table 1:** Groups of mice with different chemopreventive agent treatments.

Mouse	NTCU	Number of Mice/Group	Treated Groups	Group Symbol (Before Treatment)	Group Symbol (After Treatment)
NIH Swiss	−	6	AIN 76A diet	EbAB	EaAB
NIH Swiss	+	6	AIN 76A diet	EbD	EaD
NIH Swiss	+	6	AIN 76A diet-gavage control	EbG	EaG
NIH Swiss	+	6	Budesonide (1.5 mg/kg diet)	EbB	EaB
NIH Swiss	+	6	AZD6244 (40 mg/kg body weight-gavage)	EbA	EaA
NIH Swiss	+	6	XL-147 (100 mg/kg body weight-gavage)	EbX	EaX

**Table 2:** Differentially expressed exosomal miRNAs affected by cancer chemopreventive agent treatment.

**Differentially Expressed Exosomal miRNAs Affected by AZD6244 Treatment**						
Rank	miRNA ID	Mean EbA (log2)	Mean EaA (log2)	*p*-Value	Fold Change (log2)	False Discovery Rate (B&H)
1	mmu-miR-149-5p	8.586	6.707	0	−1.879	0.012
2	mmu-miR-24-2-5p	5.737	7.2	0	1.463	0.012
3	mmu-miR-27a-3p	9.493	10.636	0	1.143	0.012
4	mmu-miR-215-5p	9.917	11.846	0	1.929	0.023
5	mmu-miR-543-3p	12.329	11.283	0.001	−1.046	0.035
6	mmu-miR-92b-3p	11.735	11.56	0.001	−0.175	0.041
7	mmu-miR-192-5p	10.437	11.537	0.002	1.1	0.041
8	mmu-miR-744-5p	13.204	12.14	0.002	−1.064	0.041
**Differentially expressed exosomal miRNAs affected by XL-147 treatment**						
Rank	miRNA ID	Mean EbX (log2)	Mean EaX (log2)	*p*-Value	Fold Change (log2)	False Discovery Rate (B&H)
1	mmu-miR-224-5p	4.676	6.227	0	1.551	0.023
2	mmu-miR-184-3p	9.728	13.554	0	3.826	0.025
3	mmu-miR-676-3p	4.068	5.608	0	1.539	0.031
4	mmu-miR-1198-5p	7.605	8.266	0.001	0.661	0.042
**Differentially expressed exosomal miRNAs affected by Budesonide treatment**						
Rank	miRNA ID	Mean EbB (log2)	Mean EaB (log2)	*p*-Value	Fold Change (log2)	False Discovery Rate (B&H)
1	mmu-miR-378c	7.148	7.725	0	0.576	0.013

log_2_(Fold Change) = log_2_(mean read counts after treatment) − log_2_(mean read counts before treatment).

**Table 3:** Enrichment pathways of predicted target genes of four key exosomal miRNAs for AZD6244 treatment.

KEGG Pathway	Adjusted *p*-Value	#Genes	#miRNAs
Estrogen signaling pathway	4.30 × 10^−5^	12	3
Adrenergic signaling in cardiomyocytes	0.018869	15	3
Amphetamine addiction	0.04725	8	2
Lysine degradation	0.04725	4	3
AMPK signaling pathway	0.04725	12	4

**Table 4:** Enrichment pathways of predicted target genes of two key exosomal miRNAs for Budesonide treatment.

KEGG Pathway	Adjusted *p*-Value	#Genes	#miRNAs
Galactose metabolism	0.005	2	1
Mucin type O-Glycan biosynthesis	0.005	3	2
Signaling pathways regulating pluripotency of stem cells	0.019	15	2
Glycosphingolipid biosynthesis - ganglio series	0.032	3	1
Protein processing in endoplasmic reticulum	0.032	16	2
Dorso-ventral axis formation	0.032	6	2
Other glycan degradation	0.049	1	1

## Supplementary Materials

The following are available online at https://www.mdpi.com/2072-6694/11/4/477/s1, Table S1: Mapped reads of the top 100 miRNAs (log2), Table S2: Differentially expressed exosomal miRNAs affected by AZD6244 treatment, Table S3: Differentially expressed exosomal miRNAs affected by XL-147 treatment, Table S4: Differentially expressed exosomal miRNAs affected by Budesonide treatment, Table S5: Selected predictors for AZD6244, Budesonide and XL-147 treatment.

## Abbreviations and Definitions

CPA	Chemopreventive agent
DE	Differential expression
EaA	Early treatment after AZD6244
EaAB	Early treatment after absolute control
EaB	Early treatment after budesonide
EaD	Early treatment after diet control
EaG	Early treatment after early gavage control
EaX	Early treatment after XL-147
EbA	Early treatment before AZD6244
EbAB	Early treatment before absolute control
EbB	Early treatment before budesonide
EbD	Early treatment before diet control
EbG	Early treatment before early gavage control
EbX	Early treatment before XL-147
FDR	False discovery rate
Lasso	Least absolute shrinkage and selection operator
MEK	Mitogen-activated protein kinase kinase
miRNA	MicroRNA
mRNA	Messenger RNA
NTCU	N-nitroso-tris-chloroethylurea
PCA	Principal component analysis
PI3K	Phosphoinositide 3-kinase
Read Count	A term to represent the number of the RNA molecules in the RNA-sequencing libraries.
SCC	Squamous cell carcinoma
WGCNA	Weighted Gene Co-expression Network Analysis
